# Efficacy of Biomarkers in Predicting Anastomotic Leakage After Gastrointestinal Resection: A Systematic Review and Meta-Analysis

**DOI:** 10.7759/cureus.50370

**Published:** 2023-12-12

**Authors:** Khalid O Alanazi, Fahad Abdullah Alshammari, Abdulaziz S Alanazi, Muhayya Obaid Alrashidi, Ali Obaid Alrashidi, Yousif A Aldhafeeri, Tariq Hulayyil Alanazi, Abdulmalik S Alkahtani, Ahmed Sayyaf Alrakhimi, Hamdan A Albathali‏

**Affiliations:** 1 Department of General Surgery, King Khalid General Hospital, Hafar al-Batin, SAU; 2 Department of Surgery, King Khalid General Hospital, Hafar al-Batin, SAU; 3 Department of Family Medicine, Al-Shifa Primary Health Care Centre, Hafar al-Batin, SAU; 4 Department of Internal Medicine, King Khalid General Hospital, Hafar al-Batin, SAU; 5 Department of Emergency Medicine, King Khalid General Hospital, Hafar al-Batin, SAU; 6 Department of Family Medicine, Al-Nozha Primary Health Care Centre, Hafar al-Batin, SAU

**Keywords:** c-reactive protein, biomarkers, systematic review and meta-analysis, gastrointestinal cancer, anastomotic leakage

## Abstract

Our systematic review and meta-analysis were designed to evaluate the published literature from 2016 to 2019 on which the role of biomarkers in predicting the anastomotic leakage (AL) in gastroesophageal cancer surgery was investigated.

This extensive literature search was conducted on the principles of the Preferred Reporting Items for Systematic Reviews and Meta-Analyses (PRISMA) protocol. PubMed, Medical Literature Analysis and Retrieval System Online (MEDLINE), and Excerpta Medica dataBASE (EMBASE) were used to gather the relevant information. No restrictions were made on the type of biomarkers. Wald or likelihood ratio (LRT) fixed effect tests were used to estimate the pooled prevalence to generate the proportions with 95% confidence intervals (CI) and model-fitted weights. For analyzing heterogeneity, the Cochran Q test and I square test were used. The Egger regression asymmetry test and funnel plot were used for publication.

In this meta-analysis, a total of 15 studies were recruited with 1892 patients undergoing the resection. The pooled elevated C-reactive protein (CRP) was observed as 13.9% ranging from 11.6% to 16.1%. The pooled prevalence of other biomarkers with AL was observed as 4.4%. Significant heterogeneity was observed between studies that reported CRP and other biomarkers (92% each with chi-squared values of 78.80 and 122.78, respectively). However, no significant publication was observed between studies (p=0.61 and p=0.11, respectively).

We concluded our study on this note that different biomarkers are involved in the diagnosis of AL. However, all these biomarkers are poor predictors with insufficient predictive value and sensitivity.

## Introduction and background

Anastomotic leakage (AL) is one of the most common and threatening complications observed after surgical resection. This condition enhances the risk of morbidity, mortality, and prolonged hospital stay. AL also burdens the healthcare system [1-6]. Despite the advancements and availability of multimodal treatment of gastrointestinal cancers, approximately 40% of AL incidents are observed after surgical resection [6-11]. Throughout the literature, various definitions address the complication of AL [12]. Despite the extensive research on the topic, still the exact pathophysiology of AL is unclear [13-15]. Many risk factors for developing AL have been recognized [16-18], such as perioperative weight loss, blood loss, and long surgical durations [17,18]. However, it remains difficult to predict the AL in each patient individually.

During treatment duration, oral nutrition is prescribed to the patients right after day 1 of surgery. This treatment is given according to the protocol of enhanced recovery after surgery (ERAS) [19-21]. After 6-12 days of gastroesophageal resection, patients are allowed for discharge [19-21], but many cases of AL were reported in the discharge period, causing delays in diagnosis [22]. Therefore, early diagnosis of AL is necessary to avoid enhanced recovery pathways. Researchers suggested that early postoperative oral feeding should be avoided to reduce the mortality ratio in AL patients because this feeding can cause fulminant sepsis and multiple organ failures [23]. Early identification of AL cases also assists in early management with a positive effect on the quality of life and survival ratio [24].

Biomarkers are the naturally occurring indicators of normal vital living, but these can be defined as pharmacologic responses to a therapeutic intervention [25]. Various stages of ischemia, inflammations, and necrosis have been studied while investigating the biomarkers. These biomarkers also assist in predicting or diagnosing AL arising after gastroesophageal cancer surgery. In 1996, these biomarkers were studied for the first time and opened the path of extensive research. Researchers were more concerned with investigating the relationship between C-reactive protein (CRP) and intestinal cell damage markers with AL [24,25]. We designed this systematic review to evaluate the published literature from 2016 to 2019 on which the efficacy of biomarkers in evaluating the AL in gastroesophageal cancer surgery was investigated.

## Review

This extensive systematic review and meta-analysis were conducted on the principles of the Preferred Reporting Items for Systematic Reviews and Meta-Analyses (PRISMA) protocol [26]. The major aim of this study was to investigate the role of biomarkers in predicting AL after gastrointestinal resection. Databases like PubMed, Medical Literature Analysis and Retrieval System Online (MEDLINE), and Excerpta Medica dataBASE (EMBASE) were used to gather the relevant information. Two independent authors searched the literature produced from the years 2016 to 2019. Keywords like complications of anastomosis, anastomotic leakage, AND drain, marker type, biomarkers, serum, AND gastrointestinal, esophageal cancer, and upper gastrointestinal were used for finding relevant information.

Inclusion and exclusion criteria

All those studies which investigate the role of biomarkers for diagnosing AL following surgery of the esophagus or stomach were included. No restrictions were made on the type of biomarkers. Studies with sensitivity, specificity, reference standard, and clear index test were included [27,28]. We considered English-language articles with human sample sizes for inclusion while excluding previously conducted systematic reviews from our study.

Data extraction

Two authors independently evaluated the eligible studies and collected the relevant information regarding the author's name, resection type, study design, publication year, follow-up year, total sample size, AL cases, biomarker type, definition of AL, and approach. A third author resolved any disagreement with consensus.

Study outcomes

Information like the author's name, publication year, study type, total number of cases, incidents of AL, approach type, cut-off value, biomarker type, sensitivity, specificity, postoperative day (POD), positive predictive value (PPV), and negative predictive value (NPV) were extracted and described in tables.

Quality assessment or risk of bias

Two independent authors assessed the quality of included studies. For quality assessment, the Quality Assessment of Diagnostic Accuracy Studies (QUADAS) 2 tool was used. This tool comprised four main domains, including a selection of patients, index test, test timings, and reference standard.

Statistical analysis

Meta-analysis was performed by using the Meta-Mar software. Wald or likelihood ratio (LRT) fixed effect tests were used to estimate the pooled prevalence to generate the proportions with 95% confidence intervals (CI) and model-fitted weights. For analyzing heterogeneity, the Cochran Q test and I square test were used. Both tests were significant when p<0.05 and >70%, respectively. The Egger regression asymmetry test and funnel plot were used for publication bias and considered significant once the p-value was less than 0.05.

Characteristics of studies

**Figure 1:**
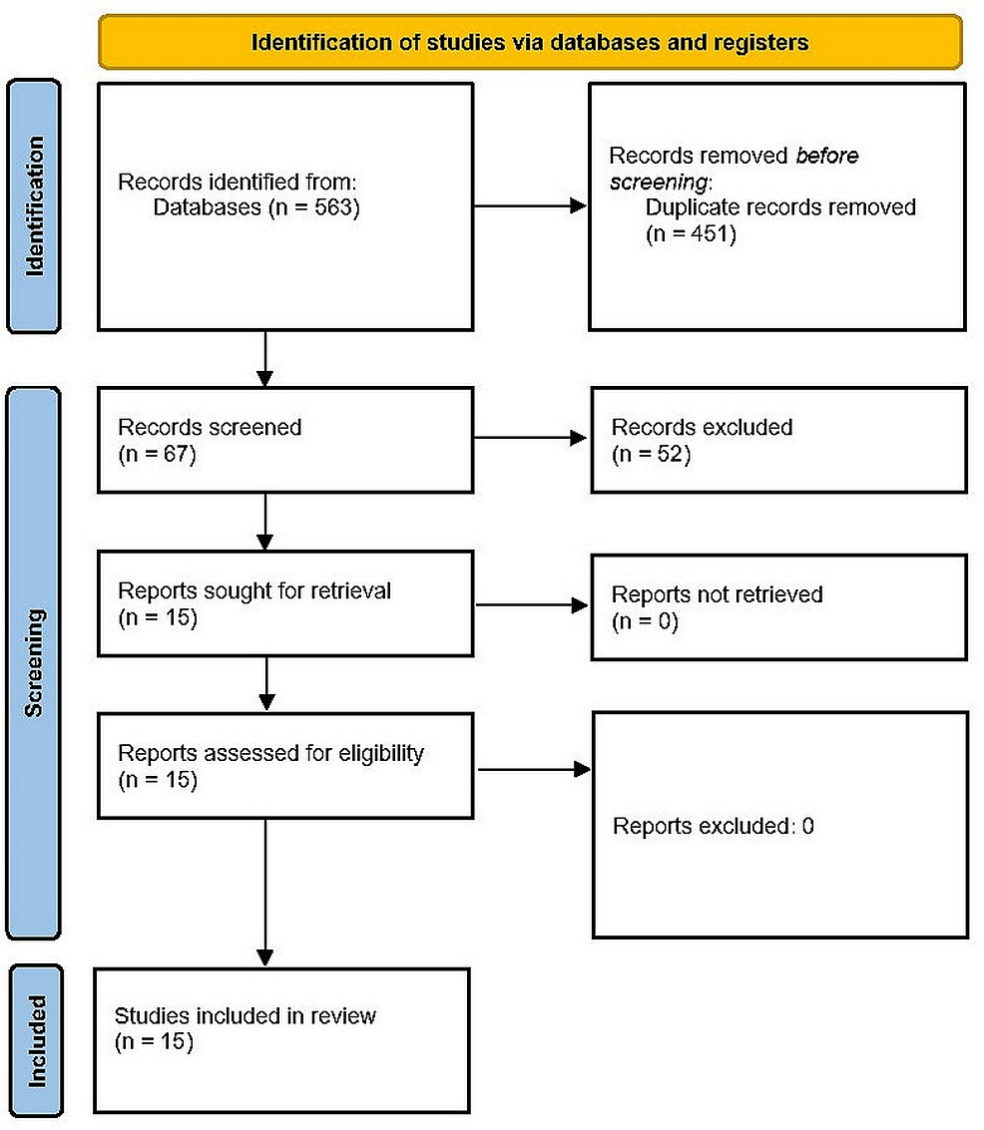
PRISMA flowchart PRISMA: Preferred Reporting Items for Systematic Reviews and Meta-Analyses

In this meta-analysis, a total of 15 studies were recruited with 1892 patients undergoing the resection (Figure 1).

All of the studies were retrospective except one. A total of 239 incidents of AL were reported during this time frame. The median POD was observed as seven days. All the selected studies were on upper gastrointestinal surgery. Four studies reported the rate of elective surgery. All of them were almost 100% elective. Overall, the rate of AL was reported at 6.7-66.2%. Various definitions of AL were identified in Table 1.

**Table 1:** Demographic characteristics of included studies CT: computed tomography; CRP: C-reactive protein; PCT: procalcitonin; Hb: hemoglobin; PLT: platelets; BG: blood G antigenemia; PO2: partial pressure of oxygen; FiO2: fraction of inspired oxygen; TNF-α: tumor necrosis factor-alpha; IL: interleukin; AL: anastomotic leakage; MI: minimally invasive; NUn: Noble and Underwood

Author (year)	Study type	Resection type	Study year	Total number of participant cases of AL (N)	Cases of AL (N)	MI approach (%)	Biomarkers	Type of marker	Defining criteria of AL
Baker et al. [28]	Retrospective	Esophageal	2009-2014	100	13	93%	Amylase and leukocytes	Serum and drain	Contrast extravasation on CT esophagram; chest CT showing empyema
Ji et al. [29]	Retrospective	Esophagogastric	2014	97	10	0%	CRP	Serum	Methylene was used orally when the fluid of abdominal drain was contaminated with blue colour than those cases defined as AL
Ip et al. [30]	Retrospective	Esophageal	2012-2014	136	18	7%	Lactate	Serum	Presence of enteric content in the chest drains along with fluoroscopy observance of oral contrast extravasation; observing esophagogastric defects on endoscopy
Paireder et al. [31]	Retrospective	Esophageal	2003-2014	258	32	28%	NUn score	Score	No proper definition; routine contrast swallow was performed
Li et al. [32]	Retrospective	Esophageal	2013-2016	71	47	8%	CRP, PCT, leukocytes, albumin, Hb, PLT, BG, PO2, and FiO2	Serum and respiratory	Esophagogastric anastomosis defects, staple line on the gastric area, or both were identified during radiological examination. Endoscopy, oral contrast CT, and methylene were used
Park et al. [33]	Retrospective	Esophageal	2009-2016	201	23	56%	CRP and leukocytes	Serum	Clinical signs; outflow of the intraluminal content due to the disruption of anastomosis; chest CT and esophagography were performed for occult leak
Song et al. [34]	Retrospective	Esophageal	2015-2016	183	16	67%	TNF-α, IL-2R, IL-6, IL-8, and IL-10	Plasma	The detection of intrathoracic findings on a CT scan, including the visualization of a contrast agent during a swallowing study and the identification of gastrointestinal tract contents through a wound or drainage tube
Asti et al. [35]	Retrospective	Esophageal	2012-2017	243	29	100%	CRP, PCT, leukocytes, and PN	Serum	Suspected by clinical signs, CT, upper gastrointestinal endoscopy for visualizing AL
Gordon et al. [36]	Retrospective	Esophagogastric	2004-2014	145	13	0%	CRP	Serum	Intraoperative AL visualized on return to theater
Schots et al. [37]	Retrospective	Gastric	2013-2017	107	8	70%	Amylase and CRP	Drain	Any signs of leakage detected on CT
Miller et al. [38]	Retrospective	Esophageal	2015-2016	45	3	100%	Amylase	Drain	No definition was used, whereas thoracic CT and fluoroscopic water-soluble method were used
Gao et al. [39]	Retrospective	Esophageal	2016-2017	96	12	100%	Amylase and prealbumin	Serum and drain	The esophagus, anastomosis, and conduit were the parameters of defining AL
Giulini et al. [40]	Retrospective	Esophageal	2015-2017	80	6	64%	Amylase and CRP	Serum and drain	Full-thickness lesion on gastric conduit; Clavien-Dindo classification grade 3 complication occurred within five days of surgery
Yu et al. [41]	Retrospective	Esophageal	2014-2017	99	10	-	Amylase	Drain	Full-thickness defect; esophagectomy was performed
Plat et al. [42]	Prospective	Esophageal	2015-2016	31	9	-	Volatile organic compounds	Urine	Clinical and radiological symptoms of full-thickness gastrointestinal defects

Approximately 22 different biomarkers were observed in selected studies. The inflammatory biomarkers including CRP, leukocytes, procalcitonin (PCT), volatile organic compounds (VOC), interleukin (IL)-2R, IL-6, IL-8, IL-10, tumor necrosis factor-alpha (TNF-α), and blood G antigenemia (BG) were observed. Elevated CRP was observed in five studies. Elevated CRP was mostly observed on day 3 of surgery. The cut-off point of CRP was observed between 83 mg/L and 299 mg/L. Meanwhile, sensitivity ranges from 55% to 100%, whereas specificity ranges from 42% to 100%.

Inflammatory biomarkers

Nine studies investigated CRP, leukocytes, PCT, albumin, prealbumin, neutrophils, VOC, IL-2R, IL-6, IL-8, IL-10, TNF-α, and BG, neutrophils, and levels of fibrinogen. One of the major and prominent inflammatory CRP markers was reported in seven studies having 128 cases of AL [29,30,32,33,35,36,38-40]. In four of these studies, a significant association was found between CRP levels and AL predictions [29,33,35,36]. In most studies, CRP levels were measured on day 3 of surgery; however, the median range of CRP was reported between one and seven days of surgery. Typically, CRP levels were elevated many days before AL diagnosis. Cut-off values of CRP were reported in a range of 83-299 mg/L at POD 1-5. The area under the curve (AUC) was observed from 0.648 to 0.994 with a sensitivity of 55-100%. A study by Ji et al. reported the highest AUC (0.994). The CRP levels were reported at 117 mg/L on the first day of surgery.

Four studies reported elevated leukocyte levels with 112 cases of AL [28,35,32,33]. A statistical significance of leukocyte was found in predicting AL. Mostly, leukocyte levels were observed on days 1-10 of surgery, but the mean duration ranges between POD 3 and 5. The AUC was found between 0.625 and 0.715. The lowest sensitivity of leukocytes was observed as 6%, with the lowest specificity as 21%.

Two studies observed elevated PCT levels measured on days 1, 3, and 5. A sufficient AUC was reported as 0.672 to very good at 0.860. Two studies revealed elevated albumin levels predicting AL cases measured on days 1-7 of surgery. The overall sensitivity was not enough ranging from 34% to 76%. A study by Gao et al. reported elevated albumin in 12 cases of AL. These levels were tested on POD 5 with a very good AUC (0.825). Asti and colleagues reported elevated percentage levels of neutrophils on POD 3, 5, and 7. A sufficient value of area under the receiver operating characteristic curve (AUROC) was reported on day 3 (0.683). This value increased on day 5 of surgery (0.692). Song et al. observed elevated cytokine levels predicting AL among 16 cases on the first day of surgery. These markers show good diagnostic accuracy (0.683 and 0.784, respectively). A study by Li et al. observed high BG in patients admitted to intensive care unit (ICU) due to respiratory distress. Out of 71 patients, these markers help diagnose AL in 47 cases.

Meta-analysis

The diverse array of biomarkers explored in these studies demonstrates their potential for predicting postoperative complications. PCT and BG, both individually and in combination, exhibited strong PPVs and NPVs, suggesting their utility in assessing postoperative risk. Additionally, IL, such as IL-10 and IL-6, displayed noteworthy sensitivity and specificity, while amylase and prealbumin contributed valuable insights into predicting complications (Table 2).

**Table 2:** Sensitivity and specificity analysis of other very good biomarkers associated with AL AL: anastomotic leakage; PPV: positive predictive value; NPV: negative predictive value; POD: postoperative day; PCT: procalcitonin; BG: blood G antigenemia; IL: interleukin; AUROC: area under the receiver operating characteristic curve; ng/mL: nanograms per milliliter; pg/mL: picograms per milliliter; IU/L: international units per liter; g/L: grams per liter

Author (year)	Biomarkers	PPV	NPV	Cut-off value	Sensitivity	Specificity	AUROC	POD
Li et al. [32]	PCT	83.3%	63.8%	3 ng/mL	72.3%	67.7%	0.752	Any
	BG	66.7%	72.3%	93 pg/mL	61.7%	83.3%	0.773	Any
	PCT×BG	91.7%	72.3%	261	72.3%	91.7%	0.773	Any
Song et al. [34]	IL-10	-	-	17.2 pg/mL	66.7%	84.8%	0.784	1
	IL-8	-	-	61.1 pg/mL	60.0%	45.7%	0.720	1
	IL-6	-	-	74.6 pg/mL	100.0%	45.7%	0.735	1
Asti et al. [35]	PCT	35%	94.2%	0.380 ng/ml	77.8%	71.4%	0.751	5
Schots et al. [37]	Amylase	31.3%	96%	750 IU/L	71.4%	81.4%	0.703	1
Giulini et al. [40]	Amylase	-	-	335 IU/L	75.0%	100%	0.814	1
Yu et al. [41]	Amylase	-	-	544 IU/L	66.7%	83.8%	0.778	3
Gao et al. [39]	Prealbumin	-	-	128 g/L	100%	50%	0.824	5

The pooled elevated CRP was observed as 13.9% ranging from 11.6% to 16.1%. The pooled prevalence of other biomarkers with AL was observed as 4.4%. A study by Li et al. and Song et al. observed three different elevated biomarkers associated with AL. In these studies, CRP levels, often with varying cut-off values, demonstrated a notable ability to predict postoperative complications, as indicated by high sensitivity and specificity values, along with AUROCs ranging from 0.8 to 0.994. CRP's PPVs varied but generally indicated its potential to identify patients at risk of complications, while high NPVs highlighted its ability to exclude complications as shown in Table 3.

**Table 3:** Sensitivity and specificity analysis of CRP biomarkers associated with AL AL: anastomotic leakage; PPV: positive predictive value; NPV: negative predictive value; POD: postoperative day; CRP: C-reactive protein; AUROC: area under the receiver operating characteristic curve; non-NT/OE: non-nuclear translocation/occupancy estimation; non-NT/MIE: non-nuclear translocation/multi-information embedding; mg/L: milligrams per liter

Author (year)	Biomarkers	PPV	NPV	Cut-off value	Sensitivity	Specificity	AUROC	POD
Ji et al. [29]	CRP	-	-	117 mg/L	90%	89%	0.994	1
		-	-	177 mg/L	90%	95%	0.908	2
		-	-	153 mg/L	90%	89%	0.936	3
		-	-	89 mg/L	90%	95%	0.917	4
		-	-	92 mg/L	90%	95%	0.881	5
Park et al. [33]	CRP (non-NT/OE)	-	-	179.4 mg/L	71.4%	72%	0.834	1
	CRP (non-NT)	-	-	171.2 mg/L	69.2%	78.1%	0.822	1
	CRP (non-NT/MIE)	-	-	128.6 mg/L	83.3%	64.9%	0.8	1
Asti et al. [35]	CRP	23.1%	97.7%	83 mg/L	89.3%	60.8%	0.818	5
Gordon et al. [36]	CRP	33.0%	100.0%	154 mg/L	100%	80%	0.907	6
		20.0%	100.0%	190 mg/L	100%	61%	0.836	3
		21.0%	100.0%	209 mg/L	100%	64%	0.819	2
Giulini et al. [40]	CRP			299 mg/L	100%	75%	0.902	2

**Figure 2:**
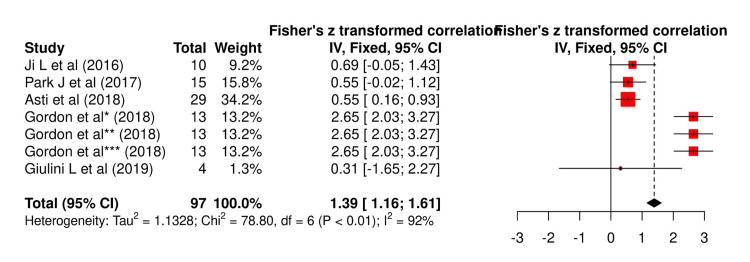
Wald fixed method of association of CRP with AL CRP: C-reactive protein, AL: anastomotic leakage References: Ji et al. [29], Park et al. [33], Asti et al. [35], Gordon et al. [36], and Giulini et al. [40]

**Figure 3:**
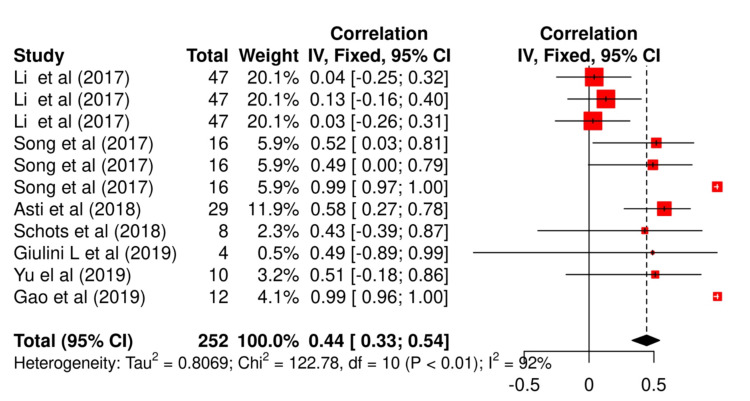
Wald fixed effect model of other biomarkers associated with AL AL: anastomotic leakage References: Li et al. [32], Song et al. [34], Asti et al. [35], Schots et al. [37], Gao et al. [39], Giulini et al. [40], and Yu et al. [41]

Significant heterogeneity was observed between studies that reported CRP and other biomarkers (92% each with chi-squared values of 78.80 and 122.78, respectively). However, no significant publication was observed between studies (p=0.61 and p=0.11, respectively) (Figure 2 and Figure 3).

Discussion

The major aim of this study was to evaluate the efficacy of biomarkers in predicting AL for cancer. We identified that systemic biomarkers, peritoneal drain fluid biomarkers, and significant high levels of mediastinal microdialysis are frequently observed in AL cases at different time points. Furthermore, upon individual assessment, it was observed that biomarkers exhibited limited diagnostic accuracy in predicting AL. However, when combined into composite scores, a notable improvement in diagnostic accuracy was observed.

The exact pathophysiology of AL is still unclear despite the extensive research conducted on animal models and humans [43,44]. Hypothetically, ischemia, inflammation, and dysbiosis were associated with AL [45]. Research also suggested that the technical aspects of surgical procedures should also be considered for defining the pathophysiology of AL [46]. Recently, two main models including the two-wound model or the two-hit hypothesis of sepsis failed to describe the exact pathophysiology of AL [47]. This research gap creates obstacles to finding new treatments or biomarkers [48]. AL can occur in the early or late stage of the post-surgical period; however, both leakages occur differently [49]. Early AL is believed to be primarily associated with technical defects during surgery, whereas late AL may occur either due to undetected early leakage or as a consequence of increased oral intake after discharge. Both leakages should be diagnosed early using the minimally invasive tool to prevent complications. Early AL, occurring shortly after surgery, is often due to technical errors during the surgical procedure. Timely diagnosis is crucial, as it allows for immediate intervention, such as reoperation or other therapeutic measures. Minimally invasive tools like diagnostic imaging and endoscopy enable the healthcare providers to identify signs of leakage, facilitating early treatment and reducing the risk of severe complications. Late AL, which can manifest weeks or months after surgery, may result from undetected early leaks or increased oral intake exerting pressure on the healing anastomosis. Diagnosis of late AL is equally vital to prevent complications such as abscess formation and sepsis. Minimally invasive diagnostic methods, including imaging and endoscopy, help assess the surgical site's integrity and detect any signs of leakage or impaired healing. Early detection of late AL allows for timely intervention, which may involve drainage, stent placement, or reoperation, as needed, to mitigate the risk of serious consequences [50].

Leakages and surgical site infections (SSIs) are the most common postoperative complications after gastrointestinal and colorectal surgery, causing pain and suffering to patients. Colorectal cancer surgery is known to carry a substantial risk of postoperative complications, with SSIs being a particularly prevalent issue. These infections can have detrimental effects on patients, leading to prolonged hospital stays, increased morbidity, a higher likelihood of readmission, and even mortality. Furthermore, the rising incidence of SSIs following both elective and urgent colorectal admissions contributes significantly to the overall cost of patient care upon hospital discharge [51]. Colorectal surgery, in particular, has been identified in the literature as posing a substantial risk for postoperative infectious complications. In addition, these complications have been associated with negative economic impact, increased morbidity, extended postoperative hospital stay, readmission, sepsis, and death. The incidence of SSIs following colorectal procedures can range from 1% to 40%, depending on the specific type of procedure performed [52]. Addressing this challenge effectively is crucial to improving patient outcomes and reducing healthcare costs associated with colorectal cancer surgery.

Microbiomes play an important role in the development of AL; however, their clear role is not completely recognized [53-55]. CRP and leukocytes are few of the major proteins which increase in response to inflammation due to infectious or noninfectious causes. Inflammatory response of resection diminished in POD 3 or 4; however, during this period, elevated CRP in patients without surgical complications indicates the presence of postoperative infectious complications [53-56]. However, CRP cannot differentiate the surgical or infectious complications since it increases in both situations [57-59]. Furthermore, CRP has a strong NPV and can prevent the occurrence of AL on POD 3-5 [35]. A previous meta-analysis of Aiolfi and colleagues stated the role of CRP in ruling out AL with reassuring clinical and radiological signs. Similarly, leukocytes as a biomarker also help in preventing AL rather than indicating this postoperative complication [60].

It was revealed that PCT is a more specific biomarker for predicting severe infection and complications. High levels of PCT indicate the presence of combined surgical and infectious complications, including AL [61-65]. However, no specific conclusion has been drawn in studies that reported high PCT levels because PCT failed to differentiate the subtypes of postoperative complications [63,64]. The study of PCT is more expensive than CRP and leukocytes, so very few laboratory tests of PCT are conducted routinely [66].

A study by Hall and colleagues evaluated the ischemic conditions of AL occurrence. They revealed that high diagnostic accuracy could be achieved by studying ischemic conditions. However, they had a small sample size and measured biomarkers involved in immune responses to many other inflammatory diseases. Along with this, one patient reported serious adverse events while placing the drain required for the dialysis and, in the end, required surgical reintervention. High levels of lactate biomarkers were also reported in the presence of ischemia [67]. These elevated levels are also one of the main factors of AL. Good diagnostic accuracy of lactate was reported in a study by Ip and colleagues for detecting the AL complication. However, this biomarker failed to account for the technical failures, and hypovolemia can influence the serum values of lactate. One study reported the Noble and Underwood (NUn) score without establishing significant external validation [31]. Several primary studies also documented the increased levels of a cost-effective and readily available biomarker, amylase, which could effectively differentiate between AL and pancreatic fistula. On the study day, Schots et al. observed significant elevation on POD 4 [28,37].

Comorbidities like diabetes mellitus, pre-existing celiac disease, and preoperative transfusion enhanced the risk of AL count. Patients with comorbidities are at high risk than the others. In high-risk patients, these risk factors should be addressed before surgery and require regular biomarker measuring [67,68]. However, in our study, no difference was observed in sensitivity and specificity among low-risk and high-risk patients. Furthermore, the affected NPVs and PPVs in high-risk patients indicate the AL complication.

## Conclusions

Our systematic review and meta-analysis contribute valuable insights into the utility of biomarkers in predicting AL following gastroesophageal cancer surgery. The observed prevalence rates, along with the acknowledgment of heterogeneity, serve as critical reference points for clinicians and researchers alike. Further exploration of this field is warranted to refine predictive models and improve patient outcomes in gastroesophageal cancer surgery. This study underscores the significance of biomarker assessment as a valuable tool in guiding clinical decision-making and advancing our understanding of postoperative complications in this context.
